# The relationship between perceived school stress and satisfaction with life among Norwegian school-based adolescents and the moderating role of perceived teacher care: a cross-sectional study

**DOI:** 10.1186/s12889-024-20246-w

**Published:** 2024-10-10

**Authors:** Erik Grasaas, Sergej Ostojic, Øyvind Sandbakk, Gunn Bjørnsen, Øystein Sylta, Daniel Høgli Major, Henriette Jahre

**Affiliations:** 1 Teacher Education Unit, University of Agder, Postbox 422, Kristiansand, 4604 Norway; 2 Department of Nutrition and Public Health, Faculty of Health and Sport Sciences, University of Agder, Kristiansand, Norway; 3 School of Sport Science, UiT The Artic University of Norway, Tromsø, Norway; 4 Kristiansand Katedralskole Gimle, Kristiansand, Norway; 5 Arendals Fysikalske, Arendal, Norway; 6 Department of Rehabilitation Science and Health Technology, Center for Intelligent Musculoskeletal Health, Oslo Metropolitan University, Oslo, Norway

**Keywords:** Stress, Satisfaction with life, Adolescents, Moderation

## Abstract

**Background:**

Perceived stress from schoolwork and perceived teacher care are shown to influence adolescents’ life satisfaction. However, there is a need to further explore how levels of perceived school stress affect life satisfaction of Norwegian adolescents across gender and school levels using nationwide data, and whether this association is moderated by perceived teacher care. Hence, this paper sought to: (1) describe perceived school stress, perceived teacher care and satisfaction with life in Norwegian adolescents stratified by gender and school level, (2) examine the association between perceived school stress and satisfaction with life by testing perceived teacher care as a possible moderator and (3) explore the association between perceived teacher care and adolescents’ satisfaction with life.

**Methods:**

We utilized cross-sectional data from the Norwegian Ungdata Survey from 2021, encompassing adolescents from lower and upper secondary school. Two-thirds of all Norwegian adolescents participated in the Ungdata Survey from 2021. All data is anonymous. The study variables are presented according to lower and upper secondary school as well as gender. Linear regressions were conducted and adjusted for socioeconomic status (SES) by using SPSS.

**Results:**

In total, 139,841 adolescents were included. Girls exhibited higher perceived school stress, lower perceived teacher care, and lower life satisfaction than boys in both lower and secondary school (all *p* < 0.01). Strong inverse associations on satisfaction with life were found in both genders in lower and upper secondary school among those who reported very frequent perceived school stress, with perceived teacher care moderating the relationship (B= -0.67; 95% CI [-0.70 to -0.65], *P* < 0.01]). Moreover, robust associations were unveiled between high and low levels of perceived teacher care and life satisfaction across gender and school level.

**Conclusions:**

Higher perceived school stress was strongly inversely associated with life satisfaction in Norwegian adolescents, in both girls and boys, and in both lower and secondary school. Teacher care was identified as a moderator and seems to play a crucial part in the everyday life of Norwegian adolescents. These implications extend to teacher education, practice, and policy, which should be aware of the pivotal role of perceived teacher care on Norwegian school-based adolescents‘ life satisfaction.

**Supplementary Information:**

The online version contains supplementary material available at 10.1186/s12889-024-20246-w.

## Background

Adolescence is a phase of physical, psychological and social processes [1]. This phase encompasses increasing demands from school that accompany individuals throughout adolescence and is suggested to be a period of heightened stress [2]. According to Lazarus and Folkman, stress is defined as “a relationship between the person and their environment that is appraised by the person as taxing or exceeding their resources and as endangering their well-being” [3]. The typical environmental context of adolescents’ lives includes several days a week of long schooldays; therefore, adolescents’ perception of school may provide essential insight into their everyday life. Research evidence suggests that stress levels among adolescents in school are even higher than in adults [4]. “The Health Behavior in School-aged Children study” with data from 2002 to 2018, including 43 countries, revealed increasing academic pressure in adolescents aged 13 and 15, and that girls, in particular, experience more stress than boys due to school pressure [5].

Findings from the Norwegian Ungdata survey have revealed an increase in perceived stress among a school-based population of adolescents, with about half of the adolescents reporting elevated levels of stress [6]. Between 2010 and 2022, a further increase in perceived stress among Norwegian school-based adolescents was revealed, constituting a public health challenge [7]. Evidence points to girls being in general more vulnerable for stress than boys in adolescence [8]. Ungdata findings have also reported higher stress related to schoolwork in girls compared to boys, with as many as two-thirds of Norwegian girls and one-third of Norwegian boys reporting being often or very often stressed by schoolwork [9].

Stress from schoolwork has been associated with psychological complaints in Norway, but was not found to be moderated by gender [10]. In 2014, Moksnes and colleagues investigated the association between school stress and life satisfaction among 13–18 year-old Norwegians and found a significant inverse association [11]. Life satisfaction is an especially interesting outcome as it is a well-known indicator of subjective well-being, providing an applicable generic score that enables comparing data across countries, times, and ages [12]. According to Diener, the measure reflects the cognitive judgment of one´s satisfaction with life [13]. Previous research has unveiled lower satisfaction with life in girls compared to boys, with an increasing difference throughout adolescence [14, 15]. According to international data from the OECD PISA report, adolescents’ mean life satisfaction scores across countries ranges between 6.1 and 8.3, with most countries scoring 7 to 7.5 out of 10 [15]. Life satisfaction among Norwegian adolescents has been reported to be reduced from 2018 to 2020 due to the heavy restrictions placed on activities under COVID-19 [16]. Research also points to other aspects important for adolescents’ satisfaction with life related to school, such as different types of teacher care [17]. Gues and colleagues reported that all kinds of teacher care, such as informational, instrumental, appraisal and emotional support were significantly associated with satisfaction with life in younger adolescents [17]. According to Teven et al., teacher care refers to an individual teacher’s behavior with care for his or her pupils [18]. Teacher care is suggested as a key factor for adolescents‘ satisfaction with life and findings indicate teacher care as a predictor for life satisfaction in adolescents [19]. McNeely and Falci explored the connection of teacher care and school connectedness and demonstrated that positive teacher-pupil relationships impact adolescents’ school engagement and reduce risky behaviors [20]. Moreover, adolescents experiencing more stress related to homework tend to experience more problems with teacher relations [21]. Yet, there is scarce evidence of how perceived teacher care potentially moderates the relationship between perceived school stress and life satisfaction in Norwegian school-based adolescents.

Therefore, there is a need for nationwide data to investigate how different levels of perceived school stress are associated with life satisfaction across gender and school levels in the school-based population of Norwegian adolescents. Findings can provide new insight for practice and policy to better understand how perceived school stress might play a key role in impacting the satisfaction of life in Norwegian school-based adolescents and potentially highlighting the pivotal role of perceived teacher care, both as a moderating role and potential independent variable. To address these current research gaps, descriptive data and association models would complement each other to enhance our understanding of how perceived teacher care impact Norwegian adolescents’ everyday life.

The aims of this paper are threefold: (1) to describe perceived school stress, perceived teacher care, and life satisfaction among Norwegian adolescents stratified by gender and school level, (2) to examine the association between perceived school stress and life satisfaction by testing perceived teacher care as possible moderator and (3) to explore the association between perceived teacher care and adolescents’ satisfaction with life.

We hypothesized that higher levels of perceived school stress are inversely associated with satisfaction with life in both lower and upper secondary school and that the relationship would be moderated by perceived teacher care. In addition, we hypothesized high perceived teacher care to be associated with higher satisfaction of life across gender and school level.

## Methods

Reporting of this study is following the Strengthening the Reporting of Observational Studies in Epidemiology (STROBE) guidelines [22]. Presented in Supplementary File 1.

### Study design

This cross-sectional study is using data from the Norwegian Ungdata Survey from 2021. The Ungdata Survey is a national survey of adolescents from lower and upper secondary school conducted every third year in almost all municipalities in Norway. The Norwegian Social Research (NOVA) at Oslo Metropolitan University and regional center for drug rehabilitation (KoRus) are responsible for the survey. The Ungdata project is financed from the national budget through grants from the Norwegian Directorate of Health [23].

### Study setting

The study takes place at school, where adolescents respond to a comprehensive electronic questionnaire during one school hour, administered by a teacher. Students not interesting in participating are given alternative school assignments. The questionnaire includes questions about the adolescents’ life, covering aspects such as well-being, lifestyle, health status, relationships, local environment, and behavior [23]. Ungdata is considered to provide a comprehensive nature of health-related data among adolescents, and thus creating opportunities of relevant public health initiatives [9].

### Participants

Norwegian adolescents from lower (13–16 years of age) and upper secondary school (16–19 years of age) are included in this study. A total of ten counties from all areas of Norway were represented in the lower secondary schools and accounted the following percent of participation: Oslo (13.8%), Rogaland (1.4%), Møre and Romsdal (4.3%), Nordland (0.2%), Viken (7.2%), Innlandet (12.3%), Vestfold and Telemark (14.7%), Vestland (22.5%), Trøndelag (15.4%) and Troms and Finnmark (8.3%). A total of eight counties, represented from all areas in Norway accounted for the following participation percent for the upper secondary schools, Oslo (13.8%), Møre and Romsdal (3.7%), Viken (9.2%), Innlandet (15.4%), Vestfold and Telemark (17.4%), Vestland (23.6%), Trøndelag (7.6%) and Troms and Finnmark (9.3%). Overall, two thirds of all Norwegian adolescents (67%) participated in the Ungdata survey in 2021 [9].

### Variables

#### Exposure

Perceived school stress was measured by using the statement “I get stressed by school work*”.* Five response alternatives were provided: “never”, “seldom”, “sometimes”, “often” and “very often”. Psychological variables from the Nordic countries have earlier been assessed to investigate the content, criterion and construct validity of single-item measures for stress, and a one-item stress-symptoms question unveiled satisfactory results of content, criterion and construct validity [24].

#### Moderator

Perceived teacher care is considered a moderator and as a possible predictor for adolescents’ satisfaction with life. The instrument is measured with the statement “My teachers care about me.” The statement had four response alternatives: “totally agree”, “somewhat agree”, “somewhat disagree”, and “totally disagree”. The response categories “totally agree” and “somewhat agree” were merged and coded as “yes” in the analysis and “somewhat disagree” and “totally disagree” were merged and coded as “no”. Perceived teacher care is treated as dichotomous variables in the analysis. This question is used in several waves of the Ungdata Survey, and in the Survey “Young in Oslo”, but is not formally validated.

#### Outcome

Satisfaction with life was assessed using the question: “On *a scale from 0 to10*,* how happy are you with your life these days?”* Higher scores indicated greater satisfaction with life. This question on satisfaction with life was originally employed in a large Norwegian study called “Young in Oslo in 2018” [25], including 25,348 adolescents. Using a single-item measure for satisfaction with life has across samples demonstrated a substantial degree of validity and performed similar to the multiple-item satisfaction with life scale [26]. Especially in adolescence, it’s reported that a single-item life satisfaction measures perform as well the satisfaction with life scale [27].

#### Covariates

Covariates used in this study are gender (boys/girls), school level (lower secondary school or higher secondary school), and socioeconomic status (SES). SES is a composite variable consisting of questions related to parental educational level, books in their home and their level of prosperity. A total sum is calculated and recoded into values from 0 to 3, of which 0 represent lowest SES and 3 the highest SES [28]. This measure is reported as a validated construct of SES [28]. As the Ungdata Survey is anonymous, data on age is not available.

#### Ethical consideration

The study is conducted in accordance with the Helsinki Declaration. Participation in the Ungdata survey is voluntary and informed written consent were provided by the adolescents. All questions from Ungdata included in this current study is approved by the Norwegian Agency for Shared Services in Education and Research (ref. 821474), known as SIKT [29]. As the survey is conducted after New Year (meaning all adolescents in upper secondary school were at least 16 years), they did not need parental consent according to Norwegian regulations. However, adolescents from lower secondary school needed a passive parental approval to participate.

### Statistical analyses

All statistical analyses were conducted using IBM SPSS Statistics for Windows, Version 25.0 (IBM Corp., Armonk, NY, USA). For the descriptive measures, continuous variables are described using means and standard deviations (SDs), and categorical variables are presented with counts and percentages. Perceived school stress, perceived teacher care and satisfaction with life are presented for the total study samples stratified by school level and gender. Chi-square tests and t-tests were used to test differences between gender for levels of perceived school stress, perceived teacher care and satisfaction with life.

Linear regressions were performed to investigate the associations between levels of perceived school stress and satisfaction with life and by exploring the association between perceived teacher care and satisfaction with life. Levels of perceived school stress and levels of perceived teacher care were created using dummy coding. Stratified regression analyses for school levels and were conducted to investigate potential age and gender differences in the associations. Crude analyses are presented, and analyses adjusted for SES.

**Fig. 1:**
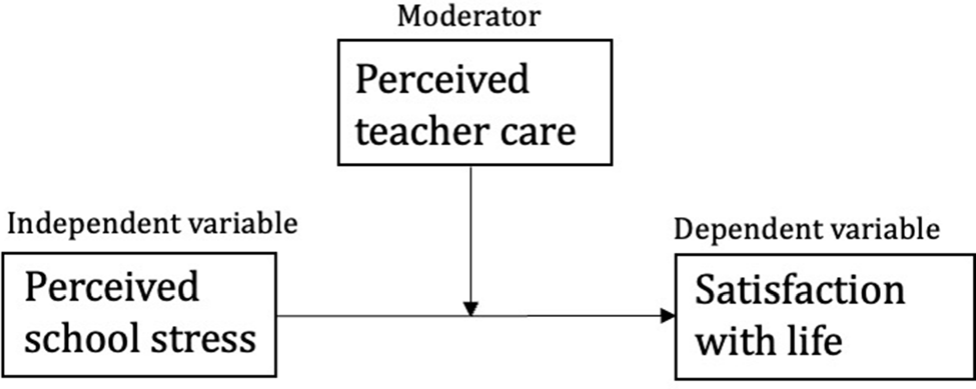
Illustration of the simple moderation analysis

To investigate whether perceived teacher care played a moderating role on the association, moderation analyses (model 1) according to Hayes using PROCESS Macro in SPSS were conducted and adjusted for SES, school level and gender. The simple moderation model is depicted in Fig. 1. Results are presented with beta coefficients with 95% confidence intervals. P-values < 0.05 were considered statistically significant, and all tests were two‐sided. Due to the high response rate, imputation was not considered needed.

## Results

### Participants

In total, 139,841 adolescents were included in the analyses, all of whom responded to the question regarding perceived school stress. Among these, 83,297 adolescents were recruited from lower secondary school and 56,544 adolescents from upper secondary school. A total of 136,498 adolescents (response rate 97.6%) responded to their assessment of satisfaction with life, and 134,272 adolescents (response rate 96.0%) responded to their assessment of perceived teacher care. The study sample exhibited nearly equal gender distribution in lower secondary school (50.7% boys and 49.3% girls) and upper secondary school (48.2% boys and 51.8% girls).

### Descriptive data of perceived school stress, perceived teacher care and satisfaction with life in Norwegian adolescents

Girls reported higher perceived school stress than boys in both lower and secondary school (*p* < 0.01) and lower satisfaction with life than boys (*p* < 0.01). Boys more frequently reported that they totally agreed that their teacher cared about them compared to girls in both lower and upper secondary school (*p* < 0.01). The distribution of perceived school stress across levels of perceived teacher care is presented in Supplementary File 2. Girls reported the same level of satisfaction with life in lower and upper secondary school with a mean of 6.7 out of 10 (Table 1). Boys reported significantly higher scores of life satisfaction in both lower and upper secondary school compared to girls, with mean scores of 7.7 and 7.3, respectively.

**Table 1:** List of study variables stratified by school level and sex

Study variables	Lower secondary school		Upper secondary school	
Perceived school stress, *n* (%) Never Seldom Sometimes Frequently Very frequently	Girls 826 (2.1%) 3302 (8.3%) 10,270 (25.7%) 10,019 (25.1%) 15,142 (37.9%)	Boys 3128 (7.6%)** 8790 (21.4%)** 13,286 (32.4%)** 7713 (18.8%)** 7202 (17.5%)**	Girls 376 (1.3%) 1484 (5.2%) 6347 (22.0%) 7709 (27.0%) 11,731 (41.1%)	Boys 2154 (8.0%)** 5123 (19.0%)** 8641 (32.0%)** 5141 (19.0%)** 4290 (15.9%)**
Perceived teacher care, n (%) Totally agree Agree to some extent Disagree to some extent Totally disagree	14,872 (37.9%) 17,964 (45.8%) 5026 (12.6%) 1328 (3.4%)	16,668 (42.0%)** 17,251 (43.5%)** 4256 (10.7%)** 1491 (3.8%)*	8567 (31.2%) 13,852 (50.5%) 4238 (15.5%) 773 (2.8%)	9591 (38.2%)** 11,957 (47.6%)** 2887 (11.5%)** 682 (2.7%)
Satisfaction with life (mean/SD) (0–10)	6.7 (2.0)	7.7 (1.8)**	6.7 (1.9)	7.3 (1.8)**

**p-value < 0.01, * p-value < 0.05

### Associations between levels of perceived school stress and satisfaction with life

Adolescents reporting that they never, seldom, or sometimes experienced school stress had a positive association with higher life satisfaction. Perceiving very frequent school stress was inversely associated with lower life satisfaction in both girls and boys in upper and lower secondary school in both crude and adjusted analyses. The inverse association for girls was stronger in lower secondary school (B = -0.17; 95% CI [-0.22 to -0.12]) than in upper secondary school (B = -0.07; 95% CI [-0.11 to -0.02]) for those reporting very frequent perceived school stress (Table 2).

**Table 2:** Crude and adjusted regression analyses of perceived school stress levels and satisfaction with life among Norwegian adolescents stratified by gender and school level

	Girls						Boys					
	Unadjusted			Adjusted			Unadjusted			Adjusted		
Lower secondary Never Seldom Sometimes Frequently Very frequently	B 1.13 0.47 0.19 -0.01 -0.16	95% CI 0.86: 1.40 0.35: 0.60 0.11: 0.27 -0.07: 0.06 -0.21: -0.11	*p-value* < 0.01 < 0.01 < 0.01 0.87 < 0.01	B 1.11 0.44 0.17 -0.02 -0.17	95% CI 0.83: 1.38 0.32: 0.57 0.09: 0.25 -0.08: 0.04 -0.22: -0.12	*p-value* < 0.01 < 0.01 < 0.01 0.50 < 0.01	B 0.86 0.35 0.13 -0.03 -0.14	95% CI 0.71: 1.00 0.28: 0.42 0.09: 0.18 -0.06: 0.01 -0.17: -0.11	*p-value* < 0.01 < 0.01 < 0.01 0.14 < 0.01	B 0.84 0.34 0.12 -0.03 -0.14	95% CI 0.69: 0.99 0.27: 0.41 0.08: 0.17 -0.07: 0.00 -0.17: -0.11	*p-value* < 0.01 < 0.01 < 0.01 0.09 < 0.01
Upper secondary Never Seldom Sometimes Frequently Very frequently	0.93 0.49 0.27 0.10 -0.05	0.65: 1.22 0.37: 0.60 0.20: 0.34 0.05: 0.15 -0.10: -0.01	< 0.01 < 0.01 < 0.01 < 0.01 < 0.05	0.95 0.47 0.25 0.08 -0.07	0.67: 1.23 0.35: 0.58 0.18: 0.32 0.03: 0.14 -0.11: -0.02	< 0.01 < 0.01 < 0.01 < 0.05 < 0.05	0.67 0.26 0.08 -0.07 -0.16	0.52: 0.82 0.19: 0.33 0.03: 0.12 -0.10: -0.03 -0.19: -0.13	< 0.01 < 0.01 < 0.01 < 0.01 < 0.01	0.69 0.26 0.08 -0.07 -0.16	0.54: 0.84 0.18: 0.33 0.03: 0.12 -0.10: -0.03 -0.19: -0.13	< 0.01 < 0.01 < 0.01 < 0.01 < 0.01

A positive association with satisfaction with life was revealed among girls who reported frequent stress in upper secondary school (B = 0.08; 95% CI [0.03 to 0.14]), which was not evident in lower secondary school nor in boys. Both genders revealed strong, robust positive associations with satisfaction with life for those reporting never, seldom, or sometimes perceived school stress, with the strongest association among girls in lower secondary school never experiencing school stress (B = 1.11; 95% CI [0.83 to 1.38]).

### The moderating role of perceived teacher care on the relationship between perceived school stress and satisfaction with life

Perceived teacher care moderated the relationship between perceived school stress and satisfaction with life for the total sample after adjusting for SES, school level, and gender (B = -0.67; 95% CI [-0.70 to -0.65], *p* < 0.01). To assess the moderation effect in the different school levels, analyses were stratified and revealed an effect modification of perceived teacher care in both lower (B = -0.73; 95% CI [-0.76 to -0.71], *p* < 0.01) and upper secondary school (B = -0.58; 95% CI [-0.62 to -0.55], *p* < 0.01) in adjusted analyses.

Associations between levels of perceived teacher care and satisfaction with life.

Regression analyses between high and low levels of perceived teacher care on satisfaction with life, stratified by gender and school level, unveiled robust findings after adjusting for SES (Table 3). Lower perceived teacher care was inversely associated with satisfaction with life, whereas higher perceived school stress was positively associated with satisfaction with life, across gender and school levels (all *p* < 0.01). For unadjusted analyses, please refer to Supplementary File 3 for details.

**Table 3:** Adjusted regressions between teacher care and satisfaction with life among Norwegian adolescents stratified by school level and gender

		Girls			Boys			
	**Lower secondary school**							
		Beta	95% CI	p value	Beta	95% CI		p value
Low perceived teacher care High perceived teacher care		-0.46 0.41	-0.55 to -0.38 0.24 to 0.57	< 0.01 < 0.01	-0.36 0.38	-0.42 to -0.30 0.27 to 0.49		< 0.01 < 0.01
	**Upper secondary school**							
		Beta	95% CI	p value	Beta	95% CI		p value
Low perceived teacher care High perceived teacher care		-0.33 0.35	-0.40 to 0.27 0.23 to 0.47	< 0.01 < 0.01	-0.24 0.47	-0.33 to -0.15 0.30 to 0.64		< 0.01 < 0.01

## Discussion

In this study, our aims were to describe perceived school stress, perceived teacher care, and satisfaction with life in Norwegian adolescents, stratified by gender and school level, and to examine the associations of perceived school stress and satisfaction with life by testing perceived teacher care as a possible moderator. Additionally, we explored the association between perceived teacher care and adolescents’ satisfaction with life. Our findings revealed that girls exhibited higher perceived school stress, lower perceived teacher care, and lower satisfaction with life than boys in both lower and upper secondary school. Regression analyses demonstrated strong inverse associations on satisfaction with life in both genders among those who reported very frequent perceived school stress, with perceived teacher care moderating the relationship. Moreover, robust associations across school levels and gender were found between high and low levels of perceived teacher care and their impact on satisfaction with life.

Our data aligns with previous international findings of lower satisfaction with life among girls compared to boys [14, 15, 30, 31], which is also the case in Norway [16, 32, 33]. As longitudinal data reports a typical decline in life satisfaction throughout adolescence [34], it was expected that boys would exhibit higher satisfaction with life in lower secondary school compared to upper secondary school, revealing a reduction in their satisfaction with life by half a point. Interestingly, our findings revealed that girls exhibited similarly relatively low levels of satisfaction with life compared to boys in both lower and upper secondary school. There might be several factors explaining the consistently lower satisfaction with life in girls. Findings from Orben et al. indicate that despite girls’ satisfaction with life being lower than boys’ in adolescence, the difference between genders does not extend into adulthood [35]. Thereby, factors might be related to girls entering puberty before boys and thus starting the decline in life satisfaction earlier than in mid-lower secondary school. Data from the International Children’s World project, including 15 countries and 48,040 participants, revealed that the tendency of lower subjective well-being starts around 10 years in most countries and that earlier puberty in girls is linked to a higher risk of reduced mental health [36, 37].

The largest discrepancies among the study variables related to gender differences were revealed by adolescents’ perceived stress. Approximately 40% of girls reported very frequent perceived stress levels compared to about 15% among boys. This pattern was evident in both lower and upper secondary school, with the highest differences observed in upper secondary school. Descriptive findings of higher stress levels in Norwegian girls are well-known from earlier Ungdata reports and other Norwegian studies [9, 11, 38]. Still, the findings of higher levels of perceived stress among girls are interesting to highlight, as research evidence indicates that higher levels of perceived stress negatively affect adolescents’ quality of life [39]. Several factors may explain the higher perceived stress level in girls than boys. According to the Norwegian report on stress and coping from the Norwegian Directorate of Health, societal trends such as very high expectations of appearance and demands for a successful life seem to influence perceived stress levels in adolescence [40]. This findings might coincide with Norwegian girls reporting doing more homework compared to boys [9], and therefore presumably perceiving more school stress due to higher expectations of appearance and demands placed on themselves compared to boys.

While the gender differences were less clear in the descriptive data of perceived teacher care, it is concerning that about one in five girls in upper secondary school disagreed to some extent or totally disagreed that their teachers cared about them. An important aspect to address here might be the adolescents’ subjective understanding of the definition of teacher care, as their understanding of what is perceived as teacher care might have influenced their responses. Research evidence points to different understandings of the term “teacher care” in early adolescence, with the majority relying on an understanding connected to kindness [41]. Lower perceived teacher care was more prominent among both genders in upper secondary school, which might be explained by the changing need for “teaching” and “care” throughout adolescence. Another aspect might be the total number of teachers during a school week. In upper secondary school, adolescents usually need to relate to a larger number of teachers compared to lower secondary school, which might also affect the perception of teacher care and the prerequisites for building solid student-teacher relations. According to a literature review by Pendergast and colleagues, a sense of belonging at school (SOBAS) might be especially important among marginalized pupils, as it is positively associated with the retention of adolescents who are at risk of dropping out [42]. Nurturing SOBAS is therefore suggested to be an aspirational goal of education [42].

As hypothesized, higher levels of perceived school stress were inversely associated with satisfaction with life in both lower and upper secondary school. These findings align with extensive research evidence of stress affecting adolescents’ subjective well-being, both nationally and internationally [38, 43–45]. As perceived school stress seems to be closely linked to adolescents’ life satisfaction, our findings of perceived teacher care moderating the relation is highly relevant due to the underpinning of the pivotal role of teachers influencing the adolescents’ life. In the study by Lavy & Naama-Ghanayim, findings also revealed that teachers’ care was associated with adolescents’ well-being [46]. However, their findings underpinned the importance of teachers’ own sense of having a meaningful work affecting their caring role. In addition, the teacher-student relation was found to mediate the relationship between teacher care and well-being [41], highlighting the complex interplay of teachers’ care and teacher-pupil relations on adolescents’ subjective well-being.

Our robust findings of perceived teacher care impacting adolescents’ lives across gender and school levels were in accordance with our hypothesis. Previous international findings have unveiled that all kinds of teacher support seem to be associated with adolescents’ satisfaction with life [17, 19]. Further, our findings correspond with earlier Norwegian studies of qualitative data, which underpin that Norwegian teachers and head teachers tend to support pupils’ mental health in their everyday practice [47]. Norwegian teachers have reported that support for pupils’ well-being is integrated into their professional identity [48]. The classroom teacher (contact teacher) has according to the Norwegian Education Act the responsibility to care for the pupils by providing a safe and sound teaching environment [49]. Adolescence seems to be a period where social support has a major impact on their everyday life. According to Sisk & Gee, the hypothalamic-pituitary-adrenal axis recalibrates during adolescence, thereby referring to adolescence as a sensitive window of opportunity, wherein social support is emphasized as particularly potent [50]. There might be several reasons why support through teacher care is important. Since adolescence is a time of identity search, teacher care might provide a sense of belonging and resilience-building in a time of new challenges. As other kinds of support, such as parental and peer support, are also linked to their well-being [51], it is natural to assume that teacher care includes both emotional support in combination with academic engagement. Teacher education needs to continue fostering competent and empathic teachers who understand their pivotal impact on Norwegian adolescents’ everyday lives.

### Strengths and limitations

This current study comprises several strengths, such as the utilization of nationwide data from Ungdata. The annual sample size was especially large in 2021 compared to earlier years due to the pandemic, which contributed to participation from all parts of Norway [9]. According to Ungdata, the non-participating municipalities do not significantly differ from the participating municipalities, thus the Ungdata 2021 survey consists of a representative population of Norwegian adolescents [9]. Moreover, a very high level of response rate (over 96%) was unveiled among the study variables. Further, Ungdata provides a validated variable for socioeconomic status [23], which should be considered a strength. Despite Ungdata being self-reported data from adolescents, which might have some uncertainties regarding the validity of the findings, 97% of adolescents in the 2021 survey reported that they answered the questions truthfully [9]. In addition, Ungdata has stringent and thorough procedures for identifying unserious responses, which further underscores the study’s credibility [23].

A clear limitation that should be highlighted stems from the non-validated categorical one-item questions used to assess perceived school stress and perceived teacher care. More comprehensive measures in these areas would have strengthened the estimates and reduced the chance of biases. Additionally, as our moderation analysis is based on theory, research evidence, and our assumptions, we have no guarantee of the direction of the associations, which should be considered as a limitation. Perceived teacher care was dichotomized in the moderation analysis, which might have reduced nuances and the variability of the study variable. Finally, as this is a cross-sectional study, extracting data from a single point in time, causality cannot be established. Since many schools were periodically closed during the COVID-19 pandemic, many adolescents were homeschooled. Thus, measures addressed in this current paper, such as school stress and teacher care, might have been influenced by this and should therefore be considered as a limitation. Given that this current dataset does not include the participating schools, adjusting for school clustering was not possible and should be considered as a limitation.

### Implications

To our knowledge, this is the first study to examine associations of perceived school stress and satisfaction with life by testing perceived teacher care as a possible moderator in Norwegian adolescents. Thus, this current study extends previous research by utilizing a large representative sample of Norwegian adolescents, controlling for relevant confounding variables, and exploring the role of teacher care in this respective relation. Moreover, our findings indicate that perceived teacher care seems to be directly linked to adolescents’ life satisfaction across gender and school level. Therefore, practice and teacher education should incorporate the importance of pupils’ perception of teacher care, as it presumably has a major impact on adolescents’ lives. Policymakers should provide frameworks allowing sufficient time for facilitating teacher care in Norwegian classrooms. Teacher education needs to continue fostering teachers with research competence who understand their pivotal role in Norwegian adolescents’ everyday lives.

## Conclusions

In this current study, girls exhibited higher perceived school stress, lower perceived teacher care, and lower satisfaction with life than boys in both lower and upper secondary school. Higher perceived school stress was strongly inversely associated with life satisfaction in Norwegian adolescents, among both girls and boys, and across both lower and upper secondary school levels. Teacher care was identified as a moderator and independent explanatory variable, indicating its crucial role in the satisfaction of life of Norwegian adolescents. The importance of teacher care should be incorporated in teacher educations and in practice by allowing sufficient time to develop positive and healthy teacher-pupil relations since this seems to moderate adolescents’ perceived stress levels. Thus, these implications extend to teacher education, practice, and policy, highlighting the importance of perceived teacher care in the life satisfaction of Norwegian school-based adolescents.

## Electronic supplementary material

Below is the link to the electronic supplementary material.

Supplementary Material 1

Supplementary File 2

Supplementary File 3

## Data Availability

The dataset that support the findings of this study is available upon request from the Norwegian Agency for Shared Services in Education and Research (SIKT) [25]. Dataset citation required from SIKT: https://doi.org/10.18712/NSD-NSD3007-V3.

## Abbreviations

CI: Confidence interval
SD: Standard deviation
SES: Socioeconomic status
NOVA: Norwegian Social Research
Korus: Regional center for drug rehabilitation
KS: The municipal sector´s organization
STROBE: Strengthening The Reporting Of Observational Studies
SIKT: Norwegian Agency for Shared Services in Education and Research
OECD: Better policies for better lives
SOBAS: A sense of belonging at school
