# Supplementary material for: The relationship between perceived school stress and satisfaction with life among Norwegian school-based adolescents and the moderating role of perceived teacher care: a cross-sectional study
Source: BMC Public Health. 2024 Oct 10;24:2771. doi: 10.1186/s12889-024-20246-w (PMC11468485; doi:10.1186/s12889-024-20246-w)
Supplement: Supplementary file 2 [file 12889_2024_20246_MOESM2_ESM.docx]

|  |  |  | | **Lower secondary school** | | | | |
| --- | --- | --- | --- | --- | --- | --- | --- | --- |
|  | | | Never stress | | Seldom stress | Sometimes stress | Often stress | Very often stress |
| Low perceived teacher care  High perceived teacher care | | | 4.1%  5.1% | | 7.2%  16.5% | 16.9%  31.7% | 21.3%  22.4% | 50.5%  24.3% |
|  |  |  | | **Upper secondary school** | | | | |
|  | | | Never stress | | Seldom stress | Sometimes stress | Often stress | Very often stress |
| Low perceived teacher care  High perceived teacher care | | | 3.4%  5.1% | | 7.0%  13.5% | 16.9%  30.4% | 21.7%  24.7% | 51.0%  26.3% |

**Supplementary File 1** Distribution of perceived school stress across levels of teacher care stratified by school levels for the total sample (i), girls (ii), and boys (iii)

1. Total sample
2. Girls

|  |  |  | | **Lower secondary school** | | | | |
| --- | --- | --- | --- | --- | --- | --- | --- | --- |
|  | | | Never stress | | Seldom stress | Sometimes stress | Often stress | Very often stress |
| Low perceived teacher care  High perceived teacher care | | | 1.6%  2.1% | | 3.8%  9.2% | 12.7%  28.6% | 21.6%  26.1% | 60.3%  34% |
|  |  |  | | **Upper secondary school** | | | | |
|  | | | Never stress | | Seldom stress | Sometimes stress | Often stress | Very often stress |
| Low perceived teacher care  High perceived teacher care | | | 1.0%  1.4% | | 2.8%  5.9% | 12.0%  25.4% | 21.5%  29.4% | 62.6%  37.9% |

1. Boys

|  |  |  | | **Lower secondary school** | | | | |
| --- | --- | --- | --- | --- | --- | --- | --- | --- |
|  | | | Never stress | | Seldom stress | Sometimes stress | Often stress | Very often stress |
| Low perceived teacher care  High perceived teacher care | | | 6.5%  8.0% | | 11.1%  23.8% | 22.4%  35.0% | 21.7%  18.8% | 38.2%  14.4% |
|  |  |  | | **Upper secondary school** | | | | |
|  | | | Never stress | | Seldom stress | Sometimes stress | Often stress | Very often stress |
| Low perceived teacher care  High perceived teacher care | | | 6.5%  8.8% | | 13.0%  21.5% | 24.2%  35.8% | 22.5%  19.9% | 33.8%  14.0% |
