# Supplementary material for: The relationship between perceived school stress and satisfaction with life among Norwegian school-based adolescents and the moderating role of perceived teacher care: a cross-sectional study
Source: BMC Public Health. 2024 Oct 10;24:2771. doi: 10.1186/s12889-024-20246-w (PMC11468485; doi:10.1186/s12889-024-20246-w)
Supplement: Supplementary file 3 [file 12889_2024_20246_MOESM3_ESM.docx]

Supplementary file 2

| **Girls Boys** | | | | | | | | |
| --- | --- | --- | --- | --- | --- | --- | --- | --- |
|  | **Lower secondary school** | | | | | |  | |
|  | | Beta | 95 % CI | p value | Beta | 95% CI | | p value |
| Low perceived teacher care  High perceived teacher care | | -0.44  0.49 | -0.52 to -0.35  0.33 to 0.66 | <0.001  <0.001 | -0.33  0.45 | -0.39 to -0.27  0.34 to 0.56 | | <0.001  <0.001 |
|  | **Upper secondary school** | | | | | |  | |
|  | | Beta | 95 % CI | p value | Beta | 95% CI | | p value |
| Low perceived teacher care  High perceived teacher care | | -0.23  0.51 | -0.32 to 0.15  0.34 to 0.68 | <0.001  <0.001 | -0.33  0.37 | -0.40 to -0.27  0.25 to 0.49 | | <0.001  <0.001 |

Unadjusted regressions between teacher care and satisfaction with life among Norwegian adolescents stratified by school level and gender
